# Abnormal structural and functional network topological properties associated with left prefrontal, parietal, and occipital cortices significantly predict childhood TBI-related attention deficits: A semi-supervised deep learning study

**DOI:** 10.3389/fnins.2023.1128646

**Published:** 2023-03-02

**Authors:** Meng Cao, Kai Wu, Jeffery M. Halperin, Xiaobo Li

**Affiliations:** ^1^Department of Biomedical Engineering, New Jersey Institute of Technology, Newark, NJ, United States; ^2^School of Biomedical Sciences and Engineering, South China University of Technology, Guangzhou, China; ^3^Department of Psychology, Queens College, City University of New York, New York, NY, United States; ^4^Department of Electrical and Computer Engineering, New Jersey Institute of Technology, Newark, NJ, United States

**Keywords:** pediatric, traumatic brain injury, attention deficits, diffusion tensor imaging, functional magnetic resonance imaging, graph theory, autoencoder, semi-supervised deep learning technique

## Abstract

**Introduction:**

Traumatic brain injury (TBI) is a major public health concern in children. Children with TBI have elevated risk in developing attention deficits. Existing studies have found that structural and functional alterations in multiple brain regions were linked to TBI-related attention deficits in children. Most of these existing studies have utilized conventional parametric models for group comparisons, which have limited capacity in dealing with large-scale and high dimensional neuroimaging measures that have unknown nonlinear relationships. Nevertheless, none of these existing findings have been successfully implemented to clinical practice for guiding diagnoses and interventions of TBI-related attention problems. Machine learning techniques, especially deep learning techniques, are able to handle the multi-dimensional and nonlinear information to generate more robust predictions. Therefore, the current research proposed to construct a deep learning model, semi-supervised autoencoder, to investigate the topological alterations in both structural and functional brain networks in children with TBI and their predictive power for post-TBI attention deficits.

**Methods:**

Functional magnetic resonance imaging data during sustained attention processing task and diffusion tensor imaging data from 110 subjects (55 children with TBI and 55 group-matched controls) were used to construct the functional and structural brain networks, respectively. A total of 60 topological properties were selected as brain features for building the model.

**Results:**

The model was able to differentiate children with TBI and controls with an average accuracy of 82.86%. Functional and structural nodal topological properties associated with left frontal, inferior temporal, postcentral, and medial occipitotemporal regions served as the most important brain features for accurate classification of the two subject groups. Post hoc regression-based machine learning analyses in the whole study sample showed that among these most important neuroimaging features, those associated with left postcentral area, superior frontal region, and medial occipitotemporal regions had significant value for predicting the elevated inattentive and hyperactive/impulsive symptoms.

**Discussion:**

Findings of this study suggested that deep learning techniques may have the potential to help identifying robust neurobiological markers for post-TBI attention deficits; and the left superior frontal, postcentral, and medial occipitotemporal regions may serve as reliable targets for diagnosis and interventions of TBI-related attention problems in children.

## 1. Introduction

Traumatic brain injury (TBI) is a major public health concern. For children in the United State, TBI-related emergency department visits exceeded 600,000 every year (Dewan et al., 2016). Children with TBI have elevated risks in developing neurocognitive impairments and behavioral abnormalities (Konigs et al., 2015; Polinder et al., 2015; Lumba-Brown et al., 2018). Significant attention deficits are among the most common cognitive consequences that can be observed in more than 35% of children two years post-TBI (Max et al., 2005). The attention problems in children post-TBI can persist into late adolescence and have been linked to the development of severe psychopathology and impairments in overall functioning (Le Fur et al., 2019; Narad et al., 2019). Without having established neurobiological signatures, treatments and interventions of TBI-related attention deficits in children have been based on subjective observations from clinicians and have resulted in suboptimal efficacy (Backeljauw and Kurowski, 2014; Kurowski et al., 2019; LeBlond et al., 2019).

In the past two decades, a number of clinical and neuroimaging studies have tried to investigate the neuroanatomical and functional substrates associated with TBI-related attention problems in children. Several diffusion tensor imaging (DTI) studies reported that the white matter integrity in corpus collosum, superior longitudinal fasciculus, and inferior fronto-occipital fasciculus were linked with impaired attention function in children with chronic TBI (Wozniak et al., 2007; Kurowski et al., 2009; Dennis et al., 2015; Konigs et al., 2018). Task-based functional magnetic resonance imaging (fMRI) studies have also reported functional alterations in frontal, parietal, and occipital regions during inhibition and sustained attention process (Kramer et al., 2008; Tlustos et al., 2011, 2015; Strazzer et al., 2015).

Known as a foundation of neuroscience, human brain regions do not work in an isolated manner. The existing voxel- and region-of-interest (ROI)-based studies have limitations in addressing how, in the systems-level, certain brain regions are vulnerable to TBI and contribute to related cognitive and behavioral consequences. The graph theoretical technique (GTT)-based approaches have been increasingly implemented in human brain imaging data to construct structural and/or functional brain networks in a systems-level, and to characterize the network integration, segregation, centrality, and small-worldness in both the global and regional (sub-network) scales (Bullmore and Sporns, 2009). Studies have reported that children with TBI demonstrated a less integrated structural or functional brain network compared to healthy controls (Caeyenberghs et al., 2012; Konigs et al., 2017; Yuan et al., 2017; Botchway et al., 2022; Ware et al., 2022). Our recent GTT-based studies in both DTI and task-based fMRI data reported that, compared to group-matched typically developing children (TDC), children with diagnosed TBI-related attention deficits (TBI-A) had significant regional topological alterations associated with frontal, parietal, and temporal lobes in both structural and functional networks, with the altered regional topological properties associated with parietal and temporal regions significantly linking to elevated inattentive symptoms in children with TBI-A (Cao et al., 2021a,b). These existing studies suggest that TBI-related attention deficits in children have close relationships with systems-level functional and structural abnormalities associated with multiple brain regions. However, all these studies have adopted conventional parametric models (such as *t*-test, analysis of variance, etc.) for group comparisons, which have very limited capacities to deal with the large-scale and nonlinearly related neuroimaging measures.

Compared to conventional parametrical models, machine learning techniques have the capacity in learning the joint effects of measures in high dimensional space and have the sensitivity in detecting subtle information that have high discriminative/predictive power (Nielsen et al., 2020). When aided with feature selection methods and cross-validation methods, machine learning techniques can deliver efficient and robust classifications between different groups. A few existing studies in children with TBI have applied machine learning techniques. By constructing classification model using support vector machine (SVM) and edge density image, one study was able to differentiate 14 children with TBI and 10 controls with an area under the receiver-operating-characteristic-curve (AUC) of 0.94 (Raji et al., 2020). Another study built an SVM-based classification model using structural MRI data and DTI data from 29 student athletes (aged from 15 to 20 years) and 27 controls and achieved an AUC of 0.84 (Tamez-Pena et al., 2021). A longitudinal study reported that when combining resting-state MRI data and structural MRI data in 99 children with TBI at 4 weeks after the injury, SVM algorithm was able to predict the recovery of post-concussion symptoms at 8 weeks with an AUC of 0.86 (Iyer et al., 2019). However, the majority of these machine learning studies in children with TBI applied supervised models that only focused on discriminating labels of the two diagnostic groups, and none of these studies have intended to detect the neurobiological features associated with the most common TBI-related cognitive deficits.

In this study, we propose to utilize a deep learning technique, semi-supervised autoencoder, to identify the robust functional and structural brain signatures of TBI-related attention deficits in children. Deep learning techniques were highly effective in generating feature representations by learning the deep linear or nonlinear relationships within a high dimensional space of the study measures (LeCun et al., 2015). Based on results of previous study from our and other teams (Wozniak et al., 2007; Kramer et al., 2008; Kurowski et al., 2009; Tlustos et al., 2011; Dennis et al., 2015; Strazzer et al., 2015; Tlustos et al., 2015; Konigs et al., 2018; Cao et al., 2021a,b), we hypothesize that topological anomalies associated with frontal, parietal, and temporal regions in the functional and structural brain networks not only play the most important role in characterizing children with TBI when compared to controls, but also most significantly contribute to TBI-related attention deficits in the affected children.

## 2. Materials and methods

### 2.1. Participants

A total of 110 children, including 55 children with TBI and 55 group-matched controls, were initially involved in this study. The TBI subjects were recruited from the New Jersey Pediatric Neuroscience Institute, Saint Peter’s University Hospital, and local communities in New Jersey. Controls were solicited from the local communities by advertisement in public places. The study received institutional review board approval at the New Jersey Institute of Technology and Saint Peter’s University Hospital. Prior the study, all the participants and their parents or guardians provided written informed assent and consent, respectively.

The inclusion criteria for the TBI group were: (1) has history of at least one clinical diagnosed mild or moderate non-penetrating TBI (Teasdale and Jennett, 1974); (2) has no overt focal brain damages or hemorrhages during all the TBI incidences; (3) the first TBI incidence was at least 6 months prior to the study date; (4) has no significant inattention or hyperactive problems before the injury. The control group included children with no history of diagnosed TBI or no history of diagnosed attention deficit/hyperactivity disorder (ADHD). Conners 3*^rd^* Edition-Parent Short form (Conners 3-PS) were assessed during the study visit to characterize the inattention problems and hyperactivity/impulsivity problems in both groups (Conners, 2008).

To further improve the homogeneity of the study sample, the general inclusion criteria for both groups included (1) only right-handed, to remove handedness-related potential effects on brain structures, which the handedness were evaluated using the Edinburgh Handedness Inventory (Oldfield, 1971); (2) full scale IQ ≥ 80, which were estimated by the Wechsler Abbreviated Scale of Intelligence II (WASI-II) (Wechsler, 2011); (3) has no current or previous diagnosis of Autism spectrum disorders, pervasive development disorder, psychosis, major mood disorders (except dysthymia not under treatment), post-traumatic stress disorder, obsessive compulsive disorder, conduct disorder, anxiety (except simple phobias), or substance use disorders, based on Diagnostic and Statistical Manual of Mental Disorders 5 (DSM-5) (Association, 2013) and supplemented by the Kiddie Schedule for Affective Disorders and Schizophrenia for School-Age Children-Present and Lifetime Version (K-SADS-PL) (Kaufman et al., 2000); (4) has no learning disabilities, neurological disorders, or any types of diagnosed chronic medical illnesses, from the medical history. None of the subjects involved in this study had any treatments with long-acting stimulants or non-stimulant psycho-tropic medications within the past month nor any contraindications for MRI scanning, such as claustrophobia, tooth braces, or other metal implants.

After initial processing of the neuroimaging data from each subject, three subjects from the TBI group and two subjects from the control group were excluded due to low imaging quality or excessive motions in either DTI data or functional MRI data. Therefore, a total of 52 children with TBI and 53 controls were included in the group-level analyses. All the demographic information was shown in Table 1.

**TABLE 1:** Demographic and clinical characteristics in the study sample.

	Controls Mean (SD)	TBI Mean (SD)	*t* or χ^2^-value	*P*- value
**N**	55	55		
**Male/female**	30/25	33/22	0.334 (χ^2^)	0.563
**Socio-economic status**	16.47 (2.13)	15.70 (2.09)	1.450	0.151
**Full scale IQ**	113.00 (11.23)	110.97 (13.72)	1.402	0.165
**Age**	13.06 (2.03)	13.63 (2.28)	−1.370	0.174
**Ethnicity/race**			4.259 (χ^2^)	0.119
Caucasian	30	36		
Hispanic	8	11		
Others	17	8		
**Conners 3rd edition-parent short form (*T*-score)**				
Inattention	46.15 (6.02)	64.73 (13.49)	−9.145	< 0.001
Hyperactivity/ impulsivity	48.38 (5.42)	58.44 (14.43)	−4.747	< 0.001

TBI, children with traumatic brain injury; SD, standard deviation; N, number of subjects; M, males; F, females.

### 2.2. Neuroimaging data acquisition protocol

For each subject, a DTI scan, a task-based functional MRI scan, and a high-resolution T1-weighed MRI scan were collected using a 3-Tesla Siemens TRIO (Siemens Medical Systems, Germany) scanner at Rutgers University Brain Imaging Center. The DTI data were acquired using a single-shot echo planar sequence, with the following parameters: voxel size = 2.0 mm × 2.0 mm × 2.5 mm, repetition time (TR) = 7,700 ms, echo time (TE) = 103 ms, field of view (FOV) = 250 mm × 250 mm, 30 diffusion-sensitizing gradient directions with *b*-value = 700 s/mm^2^, and one image with *b*-value = 0 s/mm^2^. The fMRI data were acquired using a whole brain gradient echo-planar sequence, with the following parameters: voxel size = 1.5 mm × 1.5 mm × 2.0 mm, TR = 1,000 ms, TE = 28.8 ms, and FOV = 208 mm. A high-resolution T1-weighted structural image was also collected with a sagittal multi-echo magnetization-prepared rapid acquisition gradient echo sequence with the following parameters: voxel size = 1 mm^3^ isotropic, TR = 1,900 ms, TE = 2.52 ms, flip angle = 9°, FOV = 250 mm × 250 mm, and 176 sagittal slices. The T1-weighted image were used for fMRI co-registration and creation of individualized brain region masks in DTI-based structural brain network construction.

### 2.3. Visual sustained attention task for fMRI

In the current study, the fMRI data for each subject were collected during an enhanced continuous performance task, the visual sustained attention task (VAST), which was designed to achieve optimal power in maintaining sustained attention and to assess related functional brain pathways in children (Li et al., 2012; Cao et al., 2021a). The VAST is a block-designed task which included five task stimulations block that interleaved with five resting blocks. The total duration is 5 min with each block last 30 s. During task blocks, subjects were asked to remember a sequence of three numbers and responds when the stimulus sequences match the target. To ensure full understanding of the instructions, practical trials of the task were provided to each subject before the scan session.

### 2.4. Individual level structural MRI and DTI data processing and structural brain feature generation

Each individual’s structural MRI data was visually checked for artifacts and excessive motions. Then the preprocessing steps, including registration into Talairach space, skull-stripping, and intensity normalization, were performed using Freesurfer v6.0.0 (Fischl, 2012). The preprocessed structural MRI data were parcellated using Desikan atlas and were used for node generation in constructing the structural brain network.

To construct the structural network, the DTI data were preprocessed using the Diffusion Toolbox from FMRIB Software Library v6.0 (FSL) (Woolrich et al., 2001). The preprocessing steps included head-motions correction, non-brain voxels removal, and intensities normalization. The head motions and eddy-current distortion were then corrected with affine transformation and predictions estimated by a Gaussian Process (Andersson and Sotiropoulos, 2016). Heavy head movement is a critical issue that can significantly affect the quality of imaging data and cause inaccurate results of tractography. In this study, the cutoffs of heavy head movements were defined as data with> 2 mm translational displacement, > 5° rotational displacement, or > 0.2 mm mean volume-by-volume displacement. Three subjects from TBI group and one subject from control group were excluded due to heavy head motion. Then, the probabilistic tractography parameters of each voxel were estimated with a two-fiber model in each individual’s native space. For each subject, a total of 78 ROIs were selected as the nodes for structural brain network, including 34 cortical regions and 5 subcortical regions per hemisphere. The mask for each ROI was generated based on the parcellation in the preprocessed structural MRI data and transformed into the native diffusion space. Probabilistic tractography were used to estimate the connecting fibers between each pair of the seed masks. Five thousand streamlines per voxel were then initiated from each seed mask, with 0.5 step distance. A fiber was terminated when (1) it reached other seed masks; (2) it exceeded 2,000 step limits; (3) it looped back to the same streamline; or (4) its curvature exceeded 80. The streamlines between seed masks were averaged in both directions to determine the structural connectivity between network nodes. Due to the connection density bias, the white matter bundle with higher anisotropy usually generate significantly higher streamline counts in the probabilistic tractography process (Jones, 2010; Zhang et al., 2022). Therefore, in this study, the weight of a non-zero edge was evaluated by log-transformed streamline count and normalized by dividing the maximum edge weight in the same network to increase the discriminability of low edge weights (Ashourvan et al., 2019; Hansen et al., 2022). Then for each subject, a 78 × 78 symmetric connectivity matrix was generated for construction of the weighted structural brain network.

After the weighted structural brain network was constructed for each subject, the network topological properties were calculated [technical details for computations were provided in our previous publications (Cao et al., 2021b)]. The nodal-level topological properties for weighted network, including the nodal strength, nodal global efficiency, nodal local efficiency, clustering coefficient, and betweenness centrality, were calculated for each node in the structural brain networks to serve as structural brain features. All structural network topological properties were calculated using the Brain Connectivity Toolbox (Rubinov and Sporns, 2010). A total of 390 structural brain features were generated for building the semi-supervised autoencoder.

### 2.5. Individual level fMRI data processing and functional brain feature generation

The preprocessing of the fMRI data was carried out using FEAT Toolbox from FSL v6.0 (Woolrich et al., 2001). For fMRI data, the same cutoffs of heavy head motions that used in DTI preprocessing were applied, with which two subjects from TBI group (overlapped with excluded subjects in DTI preprocessing) and one subject from control group were excluded. After motion correction and slice timing correction, the fMRI data of each subject was co-registered to standard Montreal Neurological Institute (MNI) space using high-resolution structural MRI. The hemodynamic response to the task-related condition was modeled using the general linear model with 24 motion parameters. The activated voxels were identified by cluster-based thresholding on the *Z* statistic map with *Z* > 2.3 and *p* < 0.05. To construct the functional brain network for each subject, the network nodes were generated by defining a spherical region with a radius of 5 mm at the local maximum of any clusters that have more than 100 activated voxels. A total of 59 ROIs were generated based on the automatic anatomical labeling atlas (Tzourio-Mazoyer et al., 2002). The connectivity of a ROI-pair was represented by the Pearson’s correlation coefficient of the blood-oxygen-level-dependent (BOLD) signal in each of the two ROIs. The connectivity matrix was then binarized using the network cost range that satisfied small-network property to construct the binarized functional brain network (Achard and Bullmore, 2007).

The nodal-level topological properties for binarized network, including the nodal degree, nodal global efficiency, nodal local efficiency, clustering coefficient, and betweenness centrality, were calculated for each node in the functional brain networks [technical details for computations were provided in our previous publications (Cao et al., 2021a)]. The individual-level analysis was performed using pipeline tool GAT-FD (Cao et al., 2022), where all network topological properties were calculated by calling functions from the Brain Connectivity Toolbox (Rubinov and Sporns, 2010). A total of 295 nodal topological properties were calculated from the functional brain networks to serve as the functional brain features of each subject for building the semi-supervised autoencoder.

### 2.6. Modeling of semi-supervised autoencoder

To increase training robustness and reduce overfitting risk, combination of three approaches, including two-sample *t*-test, mutual information-based method (Ross, 2014), and Lasso-based method (Muthukrishnan and Rohini, 2016), were utilized for feature reduction. At the end, a total of 60 top features from the 685 source brain features derived from structural and functional brain networks were selected for training in the model. Before passing to the autoencoder model, all these features were normalized to a range of 0 to 1 using min-max normalization.

**FIGURE 1:**
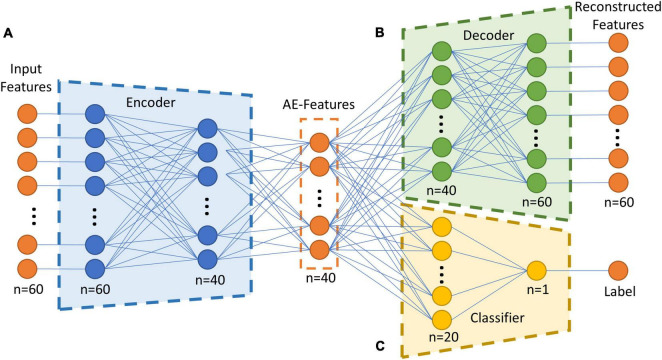
Overall structure of the semi-supervised autoencoder. **(A)** The encoder model, which transform the inputs from original brain feature space into a latent space. **(B)** The decoder model, which reconstruct the input by transforming the encoded features. **(C)** The classifier model, which predict if a subject is in the TBI group or in the control group based on the encoded features. AE-Features: autoencoder-generated features.

The semi-supervised autoencoder consisted of three major components, the encoder, the decoder, and the classifier, as shown in Figure 1. The encoder and decoder were part of a regular autoencoder model, which learns a compressed representation of the original brain features by optimizing the reconstructed brain features in an unsupervised manner (Hinton and Salakhutdinov, 2006). The encoder transformed inputs from original feature space into a latent space by compressing the information in the inputs. The encoder in the proposed model contained one input layer with a size of 60, one hidden layer of 40 neurons, and one output layer of 20 neurons. Then the autoencoder-generated features, i.e., AE-features, in the latent space were passed into the decoder to reconstruct the original input. The decoder included an input layer with a size of 20, a hidden layer of 40 neurons, and one output layer of 60 neurons. An additional classifier was included in the proposed autoencoder to work as a constrain in the learning of the compressed AE-features in the latent space. The classifier took 20 AE-features in the latent space to predict the group label for each sample. The classifier included a hidden layer with 20 neurons and an output layer of 1 neuron. Sigmoid function was used as the activation function for all the artificial neurons in the semi-supervised autoencoder neural network.

Two different loss functions were used compensate the different training speeds of the regression task (the decoder) and the classification task (the classifier). Mean squared error (MSE) was selected as the loss function of the reconstruction process, which was calculated using the following formula,


$$M\,S\,E=\frac{1}{n}\,\sum_{i=1}^{n}\left[\frac{1}{f}\,\sum_{j=1}^{f}{\left(x_{i\,j}^{\prime }-x_{i\,j}\right)}^{2}\right],$$

where *n* is the number of subjects in the training data, *f* is the number of brain features, $x_{i\,j}^{\prime }$ is the reconstructed value for feature *j* of subject *i*, and *x*_*ij*_ is original value for feature *j* of subject *i*. Binary cross-entropy were selected as the loss function of the classification process, which was calculated using the following formula,


$$H_{b\,i\,n\,a\,r\,y}=-\frac{1}{n}\,\sum_{i=1}^{n}\left[y\,\log p+\left(1-y\right)\,\log\left(1-p\right)\right],$$

where *H*_binary_ is binary cross-entropy, *n* is the number of subjects in the training data, *y* is the binary indicator of the class label, and *p* is probability of *y* is 1.

In order to force the model to learn the latent AE-features for reconstruction earlier than for classification, loss of the decoder model was assigned with a higher weight than the loss of the classifier model. The loss function of the full model was calculated using the following formula,


$$L_{f\,u\,l\,l-m\,o\,d\,e\,l}=0.7\times M\,S\,E+0.3\times H_{b\,i\,n\,a\,r\,y},$$

where the weight of the decoder loss is 0.7 and the weight of the classifier loss is 0.3.

### 2.7. Model training and evaluation

Training of the model was performed using python v3.8.0 and Tensorflow v2.10 (Abadi et al., 2016). Adam optimizer was used for the back-propagation process (Kingma and Ba, 2014). To increase the robustness of the model, a five-fold cross validation were employed in the training process. For details, the data were split into five stratified folds such that each fold consisted of balanced 20% of the entire data. For each iteration, four-folds were dedicated for training data and the remaining one for validation. To avoid potential leakage effect in the training process, the feature selection algorithms only used training data in each cross validation (Pereira et al., 2009). To further minimize the risk of overfitting in the training process, a gaussian noise with a mean of zero and standard deviation of 0.02 was randomly induced to 20% of the input features, before feeding into the encoder model. The training process stops when the accuracy of the training data exceeds 95% or reach a total of 1,000 epochs.

The performance of the reconstruction process of the semi-supervised autoencoder model were measured using the MSE of the validation data and averaged for all the five cross validations. The classification performance was measured in terms of classification accuracy and AUC in the validation data, which also averaged for all five cross validations.

In comparison, a conventional machine learning model was also constructed using the same training and validation procedure. The model used principal component analysis (PCA) for feature reduction and SVM for classification.

### 2.8. Feature importance score calculation

To identify the most important brain features for successful classification process, a permutation-based method was used to calculate the importance score of each input feature (Breiman, 2001). A feature’s importance was determined by the amount of error caused by shuffling the feature’s value over all the samples (Fisher et al., 2019). For the classification process, the feature importance for a feature was characterized by the binary cross-entropy, which was calculated using the following formula,


$$F\,I_{c\,l\,a\,s\,s}=\frac{1}{m}\,\sum_{k=1}^{m}{\left(H_{b\,i\,n\,a\,r\,y}-H_{b\,i\,n\,a\,r\,y}^{\prime }\right)}^{2},$$

where *m* is the number of random shuffling, *H*_binary_ is the cross-entropy of the original input, and $H_{\text{binary}}^{\prime }$ is the cross-entropy of the shuffled input. The importance score for features in the current study was calculated by shuffling for 1,000 times. Features with importance score that two standard deviation higher than the mean importance score of all features were identified as important features (Sun et al., 2020).

### 2.9. Modeling of brain-behavior relationships

Regression-based machine learning, a support vector regression (SVR) model, was first constructed to study the relations between the most important brain features for successful group discriminations and the severity measures of inattentive and hyperactive/impulsive symptoms (*T*-scores derived from Conners 3-PS) in the whole study sample. To minimize overfitting, five-fold cross validation were used for training and validation. The *R*^2^ and MSE were used to evaluate the performance of the SVR model. Permutation importance score were used to evaluate the importance of the brain features.

To further validate the robustness of the relationships between the identified important brain features and clinical measures, a partial least squares structural equation modeling (PLS-SEM) was conducted (Hair et al., 2011). The rationale of the PLS-SEM was to test whether the important brain features for classification were associated with any AE-features, and whether those AE-features were associated with the clinical measures, while accounting for the effects of age, sex, handedness, SES, and IQ. The PLS-SEM analysis was carried out using R 4.1.3 and SEMinR 2.3.2 (Hair et al., 2021). First, Pearson’s correlation between the AE-features in the latent space and *T*-scores of the inattentive and hyperactive/impulsive subscales from Conners 3-PS were performed within the whole study sample. The correlation analyses were controlled for potential multiple comparisons (for 20 features in the latent space), by using the Bonferroni correction with a threshold of significance at corrected α 0.05. The AE-features in the latent space that showed significant correlation with the clinical scores were selected as the intermediate variables in the PLS-SEM. Bootstrap with 5,000 random samples were performed to determine the significant levels of the path coefficients in the PLS-SEM analysis (Henseler and Chin, 2010).

## 3. Results

### 3.1. Demographic and clinical/behavioral measures

There were no significant between-group differences in any demographic measures in our sample. Among the subjects in TBI group, 14 subjects had no significant inattentive or hyperactive problems, 27 had significant inattentive problems, 2 had significant hyperactive/impulsive problems, and 12 had significant problems in both inattention and hyperactivity/impulsivity. In the TBI group, the range between first TBI incidence and MRI scan was from 6 to 90 months (7 years 6 months), with average of 33.8 ± 24.2 months. The results showed that children with TBI had significantly more inattentive (*t* = −9.145, *p* < 0.001) and hyperactive/impulsive (*t* = −4.747, *p* < 0.001) symptoms measured using the *T*-scores in Conners 3-PS, when compared to controls. No significant correlations were observed between the time after injury and inattention or hyperactivity/impulsivity *T*-scores. The demographic and clinical information was shown in Table 1.

### 3.2. Performance of the semi-supervised autoencoder

**FIGURE 2:**
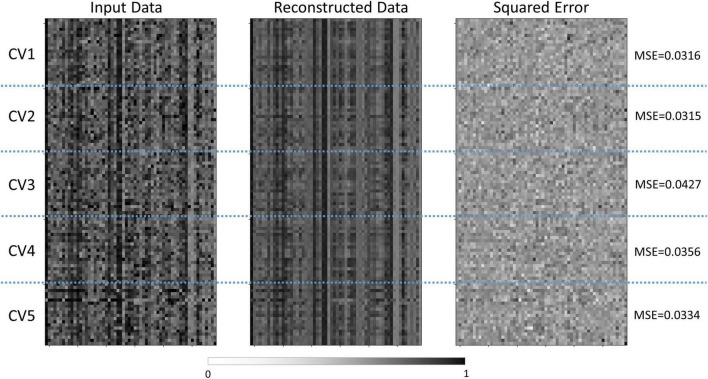
Comparisons between the normalized features and reconstructed features by the semi-supervised autoencoder model. The normalized input data was shown on the left, the reconstructed data was shown in the middle, and the squared error was shown on the right. The vertical axis represented the subjects in each cross-validation set, and the horizontal axis represented the features. CV, cross-validation; MSE, mean squared error.

The semi-supervised autoencoder model was able to differentiate children with TBI and controls with a classification accuracy of 82.86% ± 07.97% and an AUC of 0.860 ± 0.061. At the same time, the model was able to reconstruct the original brain features with an MSE of 0.035 ± 0.005, as shown in Figure 2. In comparison, the PCA+SVM model was able to achieve a classification accuracy of 78.09% ± 11.47% with an AUC of 0.825 ± 0.114.

### 3.3. Most important brain features for classification

Network topological properties associated with left inferior and superior frontal, postcentral, inferior temporal and medial occipitotemporal regions were identified as the most important brain features for successful discrimination between children with TBI and controls. Specifically, the functional nodal clustering coefficient of left inferior temporal gyrus and left medial occipitotemporal gyrus, the functional nodal local efficiency of left postcentral gyrus, the structural nodal local efficiency of left inferior frontal gyrus, the structural nodal clustering coefficient of left frontal pole, and the structural betweenness centrality of left superior frontal gyrus had significantly higher importance scores than other selected brain features (Table 2).

**TABLE 2:** Importance score of the most important brain features in accurately differentiating children with TBI and controls.

Region	Topological property	Network	Importance score
Left inferior temporal gyrus	Nodal clustering coefficient	Functional	0.0430
Left superior frontal gyrus	Betweenness centrality	Structural	0.0421
Left inferior frontal gyrus	Nodal local efficiency	Structural	0.0339
Left medial occipitotemporal gyrus	Nodal clustering coefficient	Functional	0.0338
Left postcentral gyrus	Nodal local efficiency	Functional	0.0308
Left frontal pole	Nodal clustering coefficient	Structural	0.0277

### 3.4. Regression model performance and brain-behavior relationships

**FIGURE 3:**
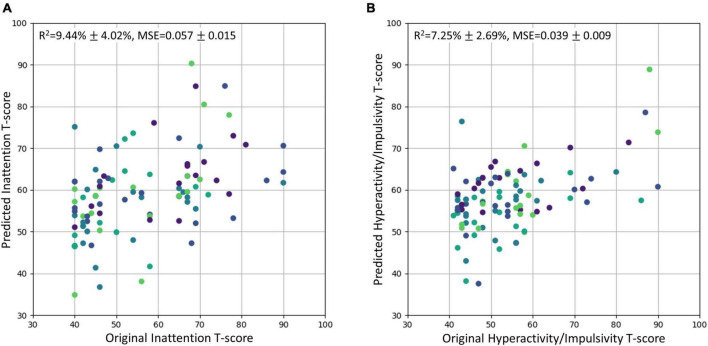
The original *T*-scores of the inattentive and hyperactive/impulsive subscales vs. the predicted *T*-scores using regression model. The predicted values were scaled back to the normal T-score range. The *R*^2^ and MSE were reported as mean ± standard deviation. Different cross-validation sets were represented with different colors. **(A)** Original inattention *T*-score vs. Predicted inattention *T*-score. **(B)** Original hyperactivity/impulsivity *T*-score vs. Predicted hyperactivity/impulsivity *T*-score. MSE, mean squared error.

The SVR model using the top 6 most important brain features was able to explain 9.44% of the variance (*R*^2^ of 9.44% ± 4.02%) in the inattentive symptom *T*-score in the study sample (Figure 3A). And the predicted inattentive symptom *T*-score yielded an MSE of 0.057 ± 0.015. The functional nodal clustering coefficient of left medial occipitotemporal gyrus and the functional nodal local efficiency of left postcentral gyrus showed the highest predictive values, with feature importance scores of 0.132 and 0.104, respectively. For the SVR model in predicting hyperactive/impulsive symptom *T*-score, the *R*^2^ was 7.25% ± 2.69% and the MSE was 0.039 ± 0.009 (Figure 3B). The most important brain features for predicting hyperactive/impulsive symptoms were the structural betweenness centrality of left superior frontal gyrus, with an importance score of 0.114, and the functional nodal clustering coefficient of left medial occipitotemporal gyrus, with an importance score of 0.050 (Table 3).

**TABLE 3:** Importance score of the most important brain features in the regression-based machine learning model for predicting inattentive and hyperactive/impulsive symptom *T*-scores in the whole study sample.

Region	Topological property	Network	Importance score
**Importance scores for predicting inattentive *T*-score**			
Left medial occipitotemporal gyrus	Nodal clustering coefficient	Functional	0.132
Left postcentral gyrus	Nodal local efficiency	Functional	0.104
Left inferior temporal gyrus	Nodal clustering coefficient	Functional	0.061
Left superior frontal gyrus	Betweenness centrality	Structural	0.014
Left frontal pole	Nodal clustering coefficient	Structural	0.013
Left inferior frontal gyrus	Nodal local efficiency	Structural	0.011
**Importance scores for predicting hyperactive/impulsive *T*-score**			
Left superior frontal gyrus	Betweenness centrality	Structural	0.114
Left medial occipitotemporal gyrus	Nodal clustering coefficient	Functional	0.050
Left inferior frontal gyrus	Nodal local efficiency	Structural	0.021
Left inferior temporal gyrus	Nodal clustering coefficient	Functional	0.017
Left postcentral gyrus	Nodal local efficiency	Functional	0.016
Left frontal pole	Nodal clustering coefficient	Structural	-0.007

**FIGURE 4:**
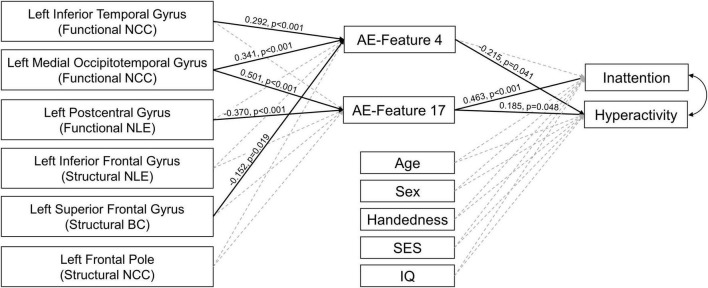
Results of partial least square structural equation modeling analysis. The paths with significant direct effects were shown in black solid line. The paths without significant effects were shown in gray dashed line. The numbers next to the significant paths were standardized path coefficient. The *p*-values were calculated by applying bootstrapping with 5,000 random samples. AE-Features: autoencoder-generated features; NCC, nodal clustering coefficient; NLE, nodal local efficiency; BC, betweenness centrality; SES, socioeconomic status, was calculated using the average education years of the parents.

In the PLM-SEM analysis, AE-feature 17 showed significant direct effect on the inattentive symptoms *T*-score, and both AE-features 4 and 17 showed significant direct effects on the hyperactive/impulsive symptoms *T*-score in the whole study sample. Important brain features in left inferior temporal, medial occipitotemporal, postcentral, and superior frontal regions showed significant direct effects on AE-features 4 and 17. The detailed results of the PLM-SEM analysis were shown in Figure 4.

## 4. Discussion

To our best knowledge, this is the first study in the field applying deep learning approach in multimodal neuroimaging data to identify the neural signatures associated with post-TBI attention deficits in children. By constructing a semi-supervised autoencoder in task-based fMRI and DTI data from 110 children, this study has identified 6 most predictive brain features, involving functional and structural network topological properties associated with left frontal, parietal, temporal, and occipital lobes. Regression-based machine learning analysis in our study sample further showed that, among these most important brain features, those associated with left postcentral area showed significant predictive value for inattentive symptoms; those associated with left superior frontal gyrus showed significant predictive value for hyperactive/impulsive symptoms; while those associated with left medial occipitotemporal gyrus showed significant predictive value for both inattentive and hyperactive/impulsive symptoms.

In the current study, our semi-supervised autoencoder model has well-behaved in terms of effectiveness and robustness in successful discrimination between children with TBI and controls, with satisfactory accuracy and AUC. The reconstructed features also showed minimal error, measured using MSE, when compared to the input features. Compared to the conventional PCA+SVM model, our semi-supervised autoencoder model achieved higher classification accuracy and AUC. The reconstruction process preserved the distinctive information while reducing the feature dimensionality for the classification process (Hinton and Salakhutdinov, 2006; Kamal and Bae, 2022). In addition, the added gaussian noise to input features during the training process of the semi-supervised autoencoder model further improve the generalization performance of the constructed deep neural network model (Audhkhasi et al., 2016; Noh et al., 2017). Therefore, relative to those reported in the majority of existing conventional model-based studies, our identified brain substrates for childhood TBI and its related attention deficits are more reliable and have more significant value in guiding tailored diagnoses and interventions in affected children.

Our study observed the important roles of the structural topological alterations of left inferior frontal gyrus, left superior frontal gyrus, and left frontal pole in differentiating children with TBI and controls. In addition, the betweenness centrality (which represent the capacity of serving as a bridging node) of left superior frontal gyrus showed significant value for successfully predicting severity of the hyperactive/impulsive symptoms in the whole study sample. Those regions were part of the prefrontal cortex, which is an essential component in the top-down control pathway that facilitate the selective attention, inhibition, and sensory modulation (Buschman and Miller, 2007; Rossi et al., 2009; Katsuki and Constantinidis, 2014). Structural MRI and DTI studies have consistently reported decreased gray matter volume, reduced cortical thickness, and disrupted white matter integrity in left prefrontal area in children with TBI (Wilde et al., 2012a; Mayer et al., 2015; Dennis et al., 2016). Our previous investigation also reported significant structural topological alterations in left inferior frontal gyrus in children with TBI-A (Cao et al., 2021b). Linking with these existing findings, our findings of altered structural connectivity within left prefrontal cortex and between left prefrontal and other brain regions may be related to the axonal damages caused TBI; and the persisted structural alterations in the left prefrontal area in children with chronic TBI might disrupt the attention processing pathways and contribute to the emergence of hyperactive/impulsive symptoms.

Meanwhile, the functional nodal local efficiency (which represent regional integration in the whole network) in the left postcentral gyrus were identified as one of the most important brain features for accurate group classification as well as one of the most valuable brain features in predicting severity of inattentive symptoms in the whole study sample. The postcentral gyrus is responsible for transferring tactile information during the spatial attention, which is a key region in the attention top-down and bottom-up pathways (Macaluso et al., 2000; Buschman and Miller, 2007; Katsuki and Constantinidis, 2014). Existing task-based fMRI studies have reported functional alterations of postcentral gyrus in children with TBI during inhibitory control (Tlustos et al., 2015) and sustained attention (Cao et al., 2021b). Our functional network study also reported that the increased nodal local efficiency in left postcentral gyrus was significantly correlated with reduced inattentive symptoms in children with TBI-A (Cao et al., 2021b). Together with existing evidence, this study further validated that functional alterations associated with left postcentral gyrus are highly vulnerable that may disrupt normal attention processing and contribute to the onset of attention deficits in children with TBI.

Intriguingly, our study also found that the functional nodal clustering coefficient (which represent the regional connectivity) in left medial occipitotemporal gyrus was an important brain feature in differentiating TBI and control, as well as a significant predictor for both inattentive and hyperactive/impulsive symptoms. The occipitotemporal gyrus has been associated with visual information processing, especially letter process (Mechelli et al., 2003; Vinckier et al., 2007), and was also found to play important role in visual imagery and internally directed cognition (Benedek et al., 2016; Ceh et al., 2021). Structural MRI studies have reported reductions in gray matter volume of the medial occipitotemporal gyrus in children with TBI, and the reduction can persist years after the injury (Wilde et al., 2012b; Dennis et al., 2016). However, no existing studies have reported functional alterations in medial occipitotemporal gyrus in children with TBI. One of the reasons might be that the conventional parametric models lack the sensitivity in detecting the subtle functional alterations in medial occipitotemporal gyrus.

There are some limitations associates with the current study. First, although we have a total of 110 subjects involved in the study, this sample size is still relatively modest in the deep learning field. Such sample size still has potential risk for having overfitted model and limited generalizability. To minimize such risk, we utilized multiple feature selection methods, applied cross-validation, and implemented an additional gaussian noise layer during the training process. Future research with an even larger sample size is expected to further validate the findings of this study. Second, streamline count-based structural brain network can be biased using probabilistic tractography (Zhang et al., 2022). To reduce potential effects, estimation of streamline count was performed in the native diffusion space using individualized brain parcellations and edge weights were normalized in the individual-level analysis. Other graph theory techniques on structural brain network, like fiber density-based (Smith et al., 2015), connectivity probability-based (Cao et al., 2013), and microstructural measure-based (Girard et al., 2017), can be explored to validate the significance of the current findings. Third, the sex factor associated with post-TBI attention deficits was not investigated in this study. Recent clinical studies with large sample size (> 500) reported that girls with TBI had significantly higher risk in developing attention problems than boys (Keenan et al., 2018; Wade et al., 2020). We did not investigate sex-specific neural markers, considering the sample size limitation mentioned above. To partially remove the potential confounding effects, sex was added in our *post hoc* analysis and showed no significant associations with inattentive or hyperactive symptoms. Future studies with much larger samples are required to thoroughly investigate the sex-specific neural markers of post-TBI attention deficits in children.

In summary, the current study has constructed a semi-supervised autoencoder to effectively and robustly discriminate children with TBI and controls while preserve the intrinsic neuroimaging characteristics in the reconstruction of brain features. All the predominant brain features in differentiating children with TBI and controls were in the left hemisphere, including the functional and structural topological alterations involving left frontal regions, postcentral regions, and temporal regions. More importantly, the highly discriminative brain features in left frontal regions, parietal regions, and medial occipitotemporal regions demonstrated significant value for predicting elevated inattentive and/or hyperactive/impulsive symptoms in children post-TBI. The findings of this study suggest that deep learning techniques may have the potential to help identifying robust neurobiological markers for post-TBI attention deficits; and the left superior frontal, postcentral, and medial occipitotemporal regions may serve as reliable targets for the diagnosis and interventions of TBI-related attention problems in children.

## Data availability statement

The raw data supporting the conclusions of this article will be made available by the authors, without undue reservation.

## Ethics statement

The studies involving human participants were reviewed and approved by Institutional Review Board at the New Jersey Institute of Technology and Institutional Review Board at Saint Peter’s University Hospital. Written informed consent to participate in this study was provided by the participants or their legal guardian/next of kin.

## Author contributions

XL and JH designed the study. MC worked on literature searching, clinical and imaging data analyses, and wrote the first draft of the manuscript. MC, KW, JH, and XL edited and revised the manuscript. All authors contributed to and have approved the final manuscript.

## References

[B1] Abadi M. Agarwal A. Barham P. Brevdo E. Chen Z. Citro C. (2016). Tensorflow: Large-scale machine learning on heterogeneous distributed systems. *arXiv [preprint]*

[B2] Achard S. Bullmore E. (2007). Efficiency and cost of economical brain functional networks. *PLoS Comput. Biol.* 3:e30017. 10.1371/journal.pcbi.0030017 17274684 PMC1794324

[B3] Andersson J. L. R. Sotiropoulos S. N. (2016). An integrated approach to correction for off-resonance effects and subject movement in diffusion MR imaging. *Neuroimage* 125 1063–1078. 10.1016/j.neuroimage.2015.10.019 26481672 PMC4692656

[B4] Ashourvan A. Telesford Q. K. Verstynen T. Vettel J. M. Bassett D. S. (2019). Multi-scale detection of hierarchical community architecture in structural and functional brain networks. *PLoS One* 14:e0215520. 10.1371/journal.pone.0215520 31071099 PMC6508662

[B5] Association A. P. (2013). *Diagnostic and statistical manual of mental disorders (DSM-5^®^).* Worcester, MA: APA. 10.1176/appi.books.9780890425596 24413388

[B6] Audhkhasi K. Osoba O. Kosko B. (2016). Noise-enhanced convolutional neural networks. *Neural Netw.* 78 15–23. 10.1016/j.neunet.2015.09.014 26700535

[B7] Backeljauw B. Kurowski B. G. (2014). Interventions for attention problems after pediatric traumatic brain injury: what is the evidence? *PM R* 6 814–824. 10.1016/j.pmrj.2014.04.004 24755513 PMC4177354

[B8] Benedek M. Jauk E. Beaty R. E. Fink A. Koschutnig K. Neubauer A. C. (2016). Brain mechanisms associated with internally directed attention and self-generated thought. *Sci. Rep.* 6:22959. 10.1038/srep22959 26960259 PMC4785374

[B9] Botchway E. Kooper C. C. Pouwels P. J. W. Bruining H. Engelen M. Oosterlaan J. (2022). Resting-state network organisation in children with traumatic brain injury. *Cortex* 154 89–104. 10.1016/j.cortex.2022.05.014 35763900

[B10] Breiman L. (2001). Random forests. *Mach. Learn.* 45 5–32. 10.1023/A:1010933404324

[B11] Bullmore E. Sporns O. (2009). Complex brain networks: graph theoretical analysis of structural and functional systems. *Nat. Rev. Neurosci.* 10 186–198. 10.1038/nrn2575 19190637

[B12] Buschman T. J. Miller E. K. (2007). Top-down versus bottom-up control of attention in the prefrontal and posterior parietal cortices. *Science* 315 1860–1862. 10.1126/science.1138071 17395832

[B13] Caeyenberghs K. Leemans A. De Decker C. Heitger M. Drijkoningen D. Linden C. V. (2012). Brain connectivity and postural control in young traumatic brain injury patients: A diffusion MRI based network analysis. *Neuroimage Clin.* 1 106–115. 10.1016/j.nicl.2012.09.011 24179743 PMC3757722

[B14] Cao M. Halperin J. M. Li X. (2021a). Abnormal functional network topology and its dynamics during sustained attention processing significantly implicate post-TBI attention deficits in children. *Brain Sci.* 11:1348. 10.3390/brainsci11101348 34679412 PMC8533973

[B15] Cao M. Luo Y. Wu Z. Mazzola C. A. Catania L. Alvarez T. L. (2021b). Topological aberrance of structural brain network provides quantitative substrates of post-traumatic brain injury attention deficits in children. *Brain Connect.* 11 651–662. 10.1089/brain.2020.0866 33765837 PMC8817712

[B16] Cao M. Wu Z. Li X. (2022). GAT-FD: An integrated MATLAB toolbox for graph theoretical analysis of task-related functional dynamics. *PLoS One* 17:e0267456. 10.1371/journal.pone.0267456 35446912 PMC9022818

[B17] Cao Q. Shu N. An L. Wang P. Sun L. Xia M. R. (2013). Probabilistic diffusion tractography and graph theory analysis reveal abnormal white matter structural connectivity networks in drug-naive boys with attention deficit/hyperactivity disorder. *J. Neurosci.* 33 10676–10687. 10.1523/JNEUROSCI.4793-12.2013 23804091 PMC6618487

[B18] Ceh S. M. Annerer-Walcher S. Koschutnig K. Korner C. Fink A. Benedek M. (2021). Neurophysiological indicators of internal attention: An fMRI-eye-tracking coregistration study. *Cortex* 143 29–46. 10.1016/j.cortex.2021.07.005 34371378

[B19] Conners C. K. (2008). *Conners 3.* Toronto, ON: MHS.

[B20] Dennis E. L. Hua X. Villalon-Reina J. Moran L. M. Kernan C. Babikian T. (2016). Tensor-based morphometry reveals volumetric deficits in moderate = severe pediatric traumatic brain injury. *J. Neurotrauma* 33 840–852. 10.1089/neu.2015.4012 26393494 PMC4860661

[B21] Dennis E. L. Jin Y. Villalon-Reina J. E. Zhan L. Kernan C. L. Babikian T. (2015). White matter disruption in moderate/severe pediatric traumatic brain injury: advanced tract-based analyses. *Neuroimage Clin.* 7 493–505. 10.1016/j.nicl.2015.02.002 25737958 PMC4338205

[B22] Dewan M. C. Mummareddy N. Wellons J. C. III Bonfield C. M. (2016). Epidemiology of global pediatric traumatic brain injury: qualitative review. *World Neurosurg.* 91:e491. 10.1016/j.wneu.2016.03.045 27018009

[B23] Fischl B. (2012). FreeSurfer. *Neuroimage* 62 774–781. 10.1016/j.neuroimage.2012.01.021 22248573 PMC3685476

[B24] Fisher A. Rudin C. Dominici F. (2019). All models are wrong, but many are useful: learning a variable’s importance by studying an entire class of prediction models simultaneously. *J. Mach. Learn. Res.* 20 1–81. 34335110 PMC8323609

[B25] Girard G. Daducci A. Petit L. Thiran J. P. Whittingstall K. Deriche R. (2017). AxTract: Toward microstructure informed tractography. *Hum. Brain Mapp.* 38 5485–5500. 10.1002/hbm.23741 28766853 PMC6866984

[B26] Hair J. F. Jr. Hult G. T. M. Ringle C. M. Sarstedt M. Danks N. P. Ray S. (2021). *Partial least squares structural equation modeling (PLS-SEM) using R: A workbook.* Berlin: Springer Nature. 10.1007/978-3-030-80519-7

[B27] Hair J. F. Ringle C. M. Sarstedt M. (2011). PLS-SEM: Indeed a silver bullet. *J. Market. Theor. Pract.* 19 139–152. 10.2753/MTP1069-6679190202

[B28] Hansen J. Y. Shafiei G. Vogel J. W. Smart K. Bearden C. E. Hoogman M. (2022). Local molecular and global connectomic contributions to cross-disorder cortical abnormalities. *Nat. Commun.* 13:4682. 10.1038/s41467-022-32420-y 35948562 PMC9365855

[B29] Henseler J. Chin W. W. (2010). A comparison of approaches for the analysis of interaction effects between latent variables using partial least squares path modeling. *Struct. Eq. Model.* 17 82–109. 10.1080/10705510903439003

[B30] Hinton G. E. Salakhutdinov R. R. (2006). Reducing the dimensionality of data with neural networks. *Science* 313 504–507. 10.1126/science.1127647 16873662

[B31] Iyer K. K. Zalesky A. Barlow K. M. Cocchi L. (2019). Default mode network anatomy and function is linked to pediatric concussion recovery. *Ann. Clin. Transl. Neurol.* 6 2544–2554. 10.1002/acn3.50951 31755665 PMC6917315

[B32] Jones D. K. (2010). Challenges and limitations of quantifying brain connectivity in vivo with diffusion MRI. *Imaging Med.* 2:341. 10.2217/iim.10.21 22281673

[B33] Kamal I. M. Bae H. (2022). Super-encoder with cooperative autoencoder networks. *Pattern Recogn.* 126:108562. 10.1016/j.patcog.2022.108562

[B34] Katsuki F. Constantinidis C. (2014). Bottom-up and top-down attention: different processes and overlapping neural systems. *Neuroscientist* 20 509–521. 10.1177/1073858413514136 24362813

[B35] Kaufman J. Birmaher B. Brent D. A. Ryan N. D. Rao U. (2000). *K-Sads-Pl.* 10.1097/00004583-200010000-00002 11026169

[B36] Keenan H. T. Clark A. E. Holubkov R. Cox C. S. Ewing-Cobbs L. (2018). Psychosocial and executive function recovery trajectories one year after pediatric traumatic brain injury: the influence of age and injury severity. *J. Neurotrauma* 35 286–296. 10.1089/neu.2017.5265 28854841 PMC5784794

[B37] Kingma D. P. Ba J. (2014). Adam: A method for stochastic optimization. *arXiv [preprint]*

[B38] Konigs M. Heij H. A. Van Der Sluijs J. A. Vermeulen R. J. Goslings J. C. Luitse J. S. (2015). Pediatric traumatic brain injury and attention deficit. *Pediatrics* 136 534–541. 10.1542/peds.2015-0437 26240208

[B39] Konigs M. Pouwels P. J. Ernest Van Heurn L. W. Bakx R. Jeroen Vermeulen R. Goslings J. C. (2018). Relevance of neuroimaging for neurocognitive and behavioral outcome after pediatric traumatic brain injury. *Brain Imaging Behav.* 12 29–43. 10.1007/s11682-017-9673-3 28092022 PMC5814510

[B40] Konigs M. Van Heurn L. W. E. Bakx R. Vermeulen R. J. Goslings J. C. Poll-The B. T. (2017). The structural connectome of children with traumatic brain injury. *Hum. Brain Mapp.* 38 3603–3614. 10.1002/hbm.23614 28429381 PMC6866988

[B41] Kramer M. E. Chiu C. Y. Walz N. C. Holland S. K. Yuan W. Karunanayaka P. (2008). Long-term neural processing of attention following early childhood traumatic brain injury: fMRI and neurobehavioral outcomes. *J. Int. Neuropsychol. Soc.* 14 424–435. 10.1017/S1355617708080545 18419841 PMC4278372

[B42] Kurowski B. Wade S. L. Cecil K. M. Walz N. C. Yuan W. Rajagopal A. (2009). Correlation of diffusion tensor imaging with executive function measures after early childhood traumatic brain injury. *J. Pediatr. Rehabil. Med.* 2 273–283. 10.3233/PRM-2009-0093 21234279 PMC3018823

[B43] Kurowski B. G. Epstein J. N. Pruitt D. W. Horn P. S. Altaye M. Wade S. L. (2019). Benefits of methylphenidate for long-term attention problems after traumatic brain injury in childhood: a randomized. double-masked, placebo-controlled, dose-titration, crossover trial. *J. Head Trauma Rehabil.* 34 E1–E12. 10.1097/HTR.0000000000000432 30169436 PMC6395577

[B44] Le Fur C. Camara-Costa H. Francillette L. Opatowski M. Toure H. Brugel D. (2019). Executive functions and attention 7years after severe childhood traumatic brain injury: Results of the Traumatisme Grave de l’Enfant (TGE) cohort. *Ann. Phys. Rehabil. Med.* 63 270–279. 10.1016/j.rehab.2019.09.003 31605766

[B45] LeBlond E. Smith-Paine J. Riemersma J. J. Horn P. S. Wade S. L. Kurowski B. G. (2019). Influence of methylphenidate on long-term neuropsychological and everyday executive functioning after traumatic brain injury in children with secondary attention problems. *J. Int. Neuropsychol. Soc.* 25 740–749. 10.1017/S1355617719000444 31178001 PMC7536786

[B46] LeCun Y. Bengio Y. Hinton G. (2015). Deep learning. *Nature* 521 436–444. 10.1038/nature14539 26017442

[B47] Li X. Sroubek A. Kelly M. S. Lesser I. Sussman E. He Y. (2012). Atypical pulvinar-cortical pathways during sustained attention performance in children with attention-deficit/hyperactivity disorder. *J. Am. Acad. Child. Adolesc. Psychiatry* 51:e1194. 10.1016/j.jaac.2012.08.013 23101745 PMC3734849

[B48] Lumba-Brown A. Yeates K. O. Sarmiento K. Breiding M. J. Haegerich T. M. Gioia G. A. (2018). Diagnosis and management of mild traumatic brain injury in children: a systematic review. *JAMA Pediatr.* 172:e182847. 10.1001/jamapediatrics.2018.2847 30193325 PMC11811836

[B49] Macaluso E. Frith C. D. Driver J. (2000). Modulation of human visual cortex by crossmodal spatial attention. *Science* 289 1206–1208. 10.1126/science.289.5482.1206 10947990

[B50] Max J. E. Schachar R. J. Levin H. S. Ewing-Cobbs L. Chapman S. B. Dennis M. (2005). Predictors of secondary attention-deficit/hyperactivity disorder in children and adolescents 6 to 24 months after traumatic brain injury. *J. Am. Acad. Child Adolesc. Psychiatry* 44 1041–1049. 10.1097/01.chi.0000173292.05817.f8 16175109

[B51] Mayer A. R. Hanlon F. M. Ling J. M. (2015). Gray matter abnormalities in pediatric mild traumatic brain injury. *J. Neurotrauma* 32 723–730. 10.1089/neu.2014.3534 25313896

[B52] Mechelli A. Gorno-Tempini M. L. Price C. J. (2003). Neuroimaging studies of word and pseudoword reading: consistencies, inconsistencies, and limitations. *J. Cogn. Neurosci.* 15 260–271. 10.1162/089892903321208196 12676063

[B53] Muthukrishnan R. Rohini R. (2016). “LASSO: A feature selection technique in predictive modeling for machine learning,” in *Proceedings of the 2016 IEEE International Conference on Advances in Computer Applications (ICACA)*, 18–20. 10.1109/ICACA.2016.7887916

[B54] Narad M. E. Riemersma J. Wade S. L. Smith-Paine J. Morrison P. Taylor H. G. (2019). Impact of Secondary ADHD on long-term outcomes after early childhood traumatic brain injury. *J. Head Trauma Rehabil.* 35 E271–E279. 10.1097/HTR.0000000000000550 31834065 PMC7205557

[B55] Nielsen A. N. Barch D. M. Petersen S. E. Schlaggar B. L. Greene D. J. (2020). Machine learning with neuroimaging: evaluating its applications in psychiatry. *Biol. Psychiatry Cogn. Neurosci. Neuroimaging* 5 791–798. 10.1016/j.bpsc.2019.11.007 31982357 PMC8746222

[B56] Noh H. You T. Mun J. Han B. (2017). Regularizing deep neural networks by noise: Its interpretation and optimization. *Adv. Neural Inform. Process. Syst.* 30 5115–5124.

[B57] Oldfield R. C. (1971). The assessment and analysis of handedness: the Edinburgh inventory. *Neuropsychologia* 9 97–113. 10.1016/0028-3932(71)90067-4 5146491

[B58] Pereira F. Mitchell T. Botvinick M. (2009). Machine learning classifiers and fMRI: a tutorial overview. *Neuroimage* 45 S199–S209. 10.1016/j.neuroimage.2008.11.007 19070668 PMC2892746

[B59] Polinder S. Haagsma J. A. Van Klaveren D. Steyerberg E. W. Van Beeck E. F. (2015). Health-related quality of life after TBI: a systematic review of study design, instruments, measurement properties, and outcome. *Popul. Health Metr.* 13:4. 10.1186/s12963-015-0037-1 25722656 PMC4342191

[B60] Raji C. A. Wang M. B. Nguyen N. Owen J. P. Palacios E. M. Yuh E. L. (2020). Connectome mapping with edge density imaging differentiates pediatric mild traumatic brain injury from typically developing controls: proof of concept. *Pediatr. Radiol.* 50 1594–1601. 10.1007/s00247-020-04743-9 32607611 PMC7501221

[B61] Ross B. C. (2014). Mutual information between discrete and continuous data sets. *PLoS One* 9:e87357. 10.1371/journal.pone.0087357 24586270 PMC3929353

[B62] Rossi A. F. Pessoa L. Desimone R. Ungerleider L. G. (2009). The prefrontal cortex and the executive control of attention. *Exp Brain Res* 192 489–497. 10.1007/s00221-008-1642-z 19030851 PMC2752881

[B63] Rubinov M. Sporns O. (2010). Complex network measures of brain connectivity: uses and interpretations. *Neuroimage* 52 1059–1069. 10.1016/j.neuroimage.2009.10.003 19819337

[B64] Smith R. E. Tournier J. D. Calamante F. Connelly A. (2015). SIFT2: Enabling dense quantitative assessment of brain white matter connectivity using streamlines tractography. *Neuroimage* 119 338–351. 10.1016/j.neuroimage.2015.06.092 26163802

[B65] Strazzer S. Rocca M. A. Molteni E. De Meo E. Recla M. Valsasina P. (2015). Altered recruitment of the attention network is associated with disability and cognitive impairment in pediatric patients with acquired brain injury. *Neural Plast.* 2015:104282. 10.1155/2015/104282 26448878 PMC4581560

[B66] Sun Y. Zhao L. Lan Z. Jia X. Z. Xue S. W. (2020). Differentiating boys with ADHD from those with typical development based on whole-brain functional connections using a machine learning approach. *Neuropsychiatr. Dis. Treat.* 16 691–702. 10.2147/NDT.S239013 32210565 PMC7071874

[B67] Tamez-Pena J. Rosella P. Totterman S. Schreyer E. Gonzalez P. Venkataraman A. (2021). Post-concussive mTBI in Student Athletes: MRI features and machine learning. *Front. Neurol.* 12:734329. 10.3389/fneur.2021.734329 35082743 PMC8784748

[B68] Teasdale G. Jennett B. (1974). Assessment of coma and impaired consciousness. A practical scale. *Lancet* 2 81–84. 10.1016/S0140-6736(74)91639-0 4136544

[B69] Tlustos S. J. Chiu C. Y. Walz N. C. Holland S. K. Bernard L. Wade S. L. (2011). Neural correlates of interference control in adolescents with traumatic brain injury: functional magnetic resonance imaging study of the counting stroop task. *J. Int. Neuropsychol. Soc.* 17 181–189. 10.1017/S1355617710001414 21092356 PMC4164964

[B70] Tlustos S. J. Peter Chiu C. Y. Walz N. C. Wade S. L. (2015). Neural substrates of inhibitory and emotional processing in adolescents with traumatic brain injury. *J. Pediatr. Rehabil. Med.* 8 321–333. 10.3233/PRM-150350 26684072 PMC5439431

[B71] Tzourio-Mazoyer N. Landeau B. Papathanassiou D. Crivello F. Etard O. Delcroix N. (2002). Automated anatomical labeling of activations in SPM using a macroscopic anatomical parcellation of the MNI MRI single-subject brain. *Neuroimage* 15 273–289. 10.1006/nimg.2001.0978 11771995

[B72] Vinckier F. Dehaene S. Jobert A. Dubus J. P. Sigman M. Cohen L. (2007). Hierarchical coding of letter strings in the ventral stream: dissecting the inner organization of the visual word-form system. *Neuron* 55 143–156. 10.1016/j.neuron.2007.05.031 17610823

[B73] Wade S. L. Kaizar E. E. Narad M. E. Zang H. Kurowski B. G. Miley A. E. (2020). Behavior problems following childhood TBI: the role of sex, age, and time since injury. *J. Head Trauma Rehabil.* 35 E393–E404. 10.1097/HTR.0000000000000567 32108717 PMC7483173

[B74] Ware A. L. Yeates K. O. Geeraert B. Long X. Beauchamp M. H. Craig W. (2022). Structural connectome differences in pediatric mild traumatic brain and orthopedic injury. *Hum. Brain Mapp.* 43 1032–1046. 10.1002/hbm.25705 34748258 PMC8764485

[B75] Wechsler D. (2011). *Wechsler Abbreviated Scale of Intelligence–Second Edition (WASI-II).* Bloomington, MN: NCS Pearson. 10.1037/t15171-000

[B76] Wilde E. A. Ayoub K. W. Bigler E. D. Chu Z. D. Hunter J. V. Wu T. C. (2012a). Diffusion tensor imaging in moderate-to-severe pediatric traumatic brain injury: changes within an 18 month post-injury interval. *Brain Imaging Behav.* 6 404–416. 10.1007/s11682-012-9150-y 22399284

[B77] Wilde E. A. Merkley T. L. Bigler E. D. Max J. E. Schmidt A. T. Ayoub K. W. (2012b). Longitudinal changes in cortical thickness in children after traumatic brain injury and their relation to behavioral regulation and emotional control. *Int. J. Dev. Neurosci.* 30 267–276. 10.1016/j.ijdevneu.2012.01.003 22266409 PMC3322311

[B78] Woolrich M. W. Ripley B. D. Brady M. Smith S. M. (2001). Temporal autocorrelation in univariate linear modeling of FMRI data. *Neuroimage* 14 1370–1386. 10.1006/nimg.2001.0931 11707093

[B79] Wozniak J. R. Krach L. Ward E. Mueller B. A. Muetzel R. Schnoebelen S. (2007). Neurocognitive and neuroimaging correlates of pediatric traumatic brain injury: a diffusion tensor imaging (DTI) study. *Arch. Clin. Neuropsychol.* 22 555–568. 10.1016/j.acn.2007.03.004 17446039 PMC2887608

[B80] Yuan W. Treble-Barna A. Sohlberg M. M. Harn B. Wade S. L. (2017). Changes in structural connectivity following a cognitive intervention in children with traumatic brain injury. *Neurorehabil. Neural. Repair.* 31 190–201. 10.1177/1545968316675430 27798379

[B81] Zhang F. Daducci A. He Y. Schiavi S. Seguin C. Smith R. E. (2022). Quantitative mapping of the brain’s structural connectivity using diffusion MRI tractography: A review. *Neuroimage* 249:118870. 10.1016/j.neuroimage.2021.118870 34979249 PMC9257891

